# Hsp90 inhibition protects against inherited retinal degeneration

**DOI:** 10.1093/hmg/ddt613

**Published:** 2013-12-02

**Authors:** Mònica Aguilà, Dalila Bevilacqua, Caroline McCulley, Nele Schwarz, Dimitra Athanasiou, Naheed Kanuga, Sergey S. Novoselov, Clemens A.K. Lange, Robin R. Ali, James W. Bainbridge, Carlos Gias, Peter J. Coffey, Pere Garriga, Michael E. Cheetham

**Affiliations:** 1 Department of Ocular Biology and Therapeutics,; 2 Department of Genetics, UCL Institute of Ophthalmology, 11–43 Bath Street, London EC1V 9EL, UK; 3 Centre de Biotecnologia Molecular, Departament d'Enginyeria Química, Universitat Politècnica de Catalunya, Rambla Sant Nebridi 22, 08222 Terrassa, Catalonia, Spain

## Abstract

The molecular chaperone Hsp90 is important for the functional maturation of many client proteins, and inhibitors are in clinical trials for multiple indications in cancer. Hsp90 inhibition activates the heat shock response and can improve viability in a cell model of the P23H misfolding mutation in rhodopsin that causes autosomal dominant retinitis pigmentosa (adRP). Here, we show that a single low dose of the Hsp90 inhibitor HSP990 enhanced visual function and delayed photoreceptor degeneration in a P23H transgenic rat model. This was associated with the induction of heat shock protein expression and reduced rhodopsin aggregation. We then investigated the effect of Hsp90 inhibition on a different type of rod opsin mutant, R135L, which is hyperphosphorylated, binds arrestin and disrupts vesicular traffic. Hsp90 inhibition with 17-AAG reduced the intracellular accumulation of R135L and abolished arrestin binding in cells. *Hsf-1*^−/−^ cells revealed that the effect of 17-AAG on P23H aggregation was dependent on HSF-1, whereas the effect on R135L was HSF-1 independent. Instead, the effect on R135L was mediated by a requirement of Hsp90 for rhodopsin kinase (GRK1) maturation and function. Importantly, Hsp90 inhibition restored R135L rod opsin localization to wild-type (WT) phenotype *in vivo* in rat retina. Prolonged Hsp90 inhibition with HSP990 *in vivo* led to a posttranslational reduction in GRK1 and phosphodiesterase (PDE6) protein levels, identifying them as Hsp90 clients. These data suggest that Hsp90 represents a potential therapeutic target for different types of rhodopsin adRP through distinct mechanisms, but also indicate that sustained Hsp90 inhibition might adversely affect visual function.

## INTRODUCTION

Hsp90 is an abundant and highly conserved molecular chaperone that is involved in many cellular processes, including the functional maturation of substrate proteins, which are known as ‘clients’ (1,2). Several of these client proteins are oncogenes, leading to Hsp90 emerging as an important target in different types of cancer treatment (3). Nucleotide binding and posttranslational modifications regulate Hsp90 function (4). Hsp90 inhibitors bind with a high affinity to the ATP-binding pocket and block the chaperone ATPase cycle leading to the degradation of client proteins (2,3). Inhibition of Hsp90 function also disrupts the chaperone complex with Heat Shock Factor 1 (HSF-1), causing the activation of HSF-1 and induction of heat shock protein expression (5). Therefore, Hsp90 inhibition can elicit a dual effect, the proteasome-mediated degradation of Hsp90 client proteins and activation of HSF-1, which induces Hsp70 and other chaperones to protect against protein aggregation and reduce protein toxicity (6–8).

Retinitis pigmentosa (RP) is the most common form of inherited photoreceptor degeneration. RP leads to dysfunction and progressive loss of photoreceptor cells, resulting in defective dark adaptation, reduction of peripheral vision and ultimately blindness (9). Mutations in the rhodopsin gene, *RHO*, are the most common cause of autosomal dominant RP (adRP) (10) (RetNet: http://www.sph.uth.tmc/edu/Retnet/). Rhodopsin, the light sensitive protein of rod cells comprises rod opsin protein and chromophore 11-*cis*-retinal. Over 150 mutations in rod opsin result in different cellular and biochemical defects, such as protein misfolding, defective chromophore binding, impaired G-protein coupling or activation, and altered traffic (11). Pharmacological interventions can improve protein folding, reduce aggregation and improve the traffic of the P23H Class II misfolding rod opsin mutant (12). For example, treatment with Hsp90 inhibitors, including the geldanamycin analog 17-allylamino-17-demethoxygeldanamycin (17-AAG), led to a reduction in P23H protein aggregation that correlated with an increase in cell viability (12), highlighting the potential of Hsp90 inhibitors to slow inherited retinal degeneration associated with defects in photoreceptor proteostasis (13).

Amino acid substitutions at arginine 135 of rod opsin (e.g. R135L), which is highly conserved in GPCRs, cause a severe and fast progressing form of adRP (14,15). The R135L mutant is constitutively phosphorylated and binds with a high affinity to visual arrestin (16), recruiting and translocating cytosolic arrestin to the plasma membrane (PM) and endocytic compartments (14). As yet, pharmacological interventions for this class of rod opsin mutant have not been investigated. In the present study, we investigated the therapeutic potential of Hsp90 inhibition to reduce P23H rod opsin associated cell death in the retina. We also tested the ability of pharmacological chaperones, kosmostropes and Hsp90 inhibitors to alleviate the R135L rod opsin phenotype. The results identify distinct mechanisms for the therapeutic potential of Hsp90 inhibitors in retinal degeneration, but also reveal the role of Hsp90 in the maturation of essential phototransduction components.

## RESULTS

### HSP990-mediated protection of photoreceptor function and survival in P23H-1 rats

**Figure 1.**
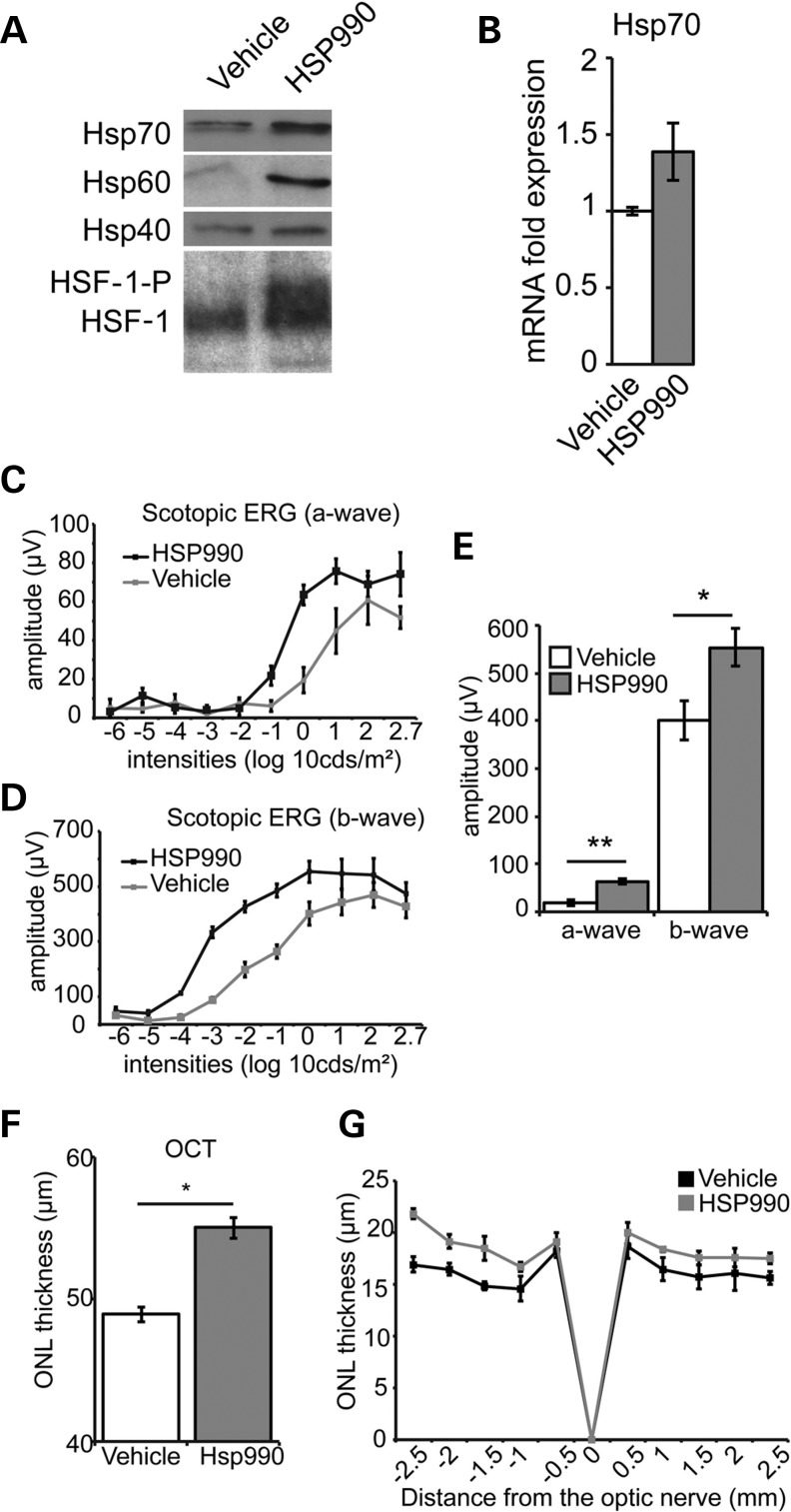
HSP990 protects photoreceptor function and survival in P23H-1 rats. (**A**) Western blots of the HSR in mouse retina systemically treated with HSP990 (20 mg/kg) or vehicle. Retinae were collected 24 h postadministration and 10 μg total protein western blotted for Hsp70, Hsp60 or Hsp40, as indicated. For the western blot for HSF-1, retinae were harvested 2 h after HSP990 (20 mg/kg) or vehicle administration. Posttranslational modification of HSF-1 such as phosphorylation is indicated (HSF-1-P). (**B**) RT–PCR showing retinal Hsp70 mRNA induction 2 h after HSP990 administration. Scotopic ERG responses, a-wave (**C**), b-wave (**D**) and average at 1 log 10 cds/m^2^, (**E**) in vehicle or HSP990-treated P23H-1 rats at P35, following a single-dose HSP990 treatment at P21. *P ≤ 0.05, values are means ± SEM, *n* ≥ 5 (**F**) P23H-1 ONL thickness at P35 after a single dose of HSP990 at P21 assessed by OCT measurements. *P ≤ 0.05, values are means ± SEM, *n* ≥ 4. (**G**) Spider plot of ONL thickness in vehicle and HSP990-treated animals at P35 following a single treatment at 21 days old. *P ≤ 0.05, values are mean ± SEM (*n* ≥ 5 per treatment group).

We have previously shown that 17-AAG could protect against P23H rod opsin in cells (12); however, 17-AAG does not efficiently cross the blood retinal barrier (17). The Hsp90 inhibitor 2-amino-7,8-dihydro-6H-pyrido[4,3-d]pyrimidin-5-one NVP-HSP990 (referred to herein as HSP990) is a potent inducer of the heat shock response (HSR) in brain (8), so we reasoned that it was likely to cross the blood retinal barrier and would be more appropriate to study the effect of Hsp90 inhibition on the retina *in vivo*. Systemic HSP990 treatment led to the rapid posttranslational modification of HSF-1 in mouse retina, as judged by its reduced SDS–PAGE mobility (Fig. 1A). Retinal Hsp70 mRNA was increased (Fig. 1B), and Hsp70, Hsp60 and Hsp40 protein levels were also increased (Fig. 1A). These results show that HSP990 is able to efficiently induce the HSR in retina *in vivo*, confirming that it can cross the blood retinal barrier.

Transgenic P23H-1 rats that express P23H rod opsin in their photoreceptors and undergo rapid and progressive photoreceptor degeneration (18) were treated with a single dose of HSP990 at 21 days of age (P21) when the degeneration is already established. Full-field scotopic electroretinogram (ERG) was performed 14 days later (P35) to assess changes in retinal function. ERG analysis showed that HSP990 treatment preserved photoreceptor activity in P23H-1 rats, as the a-wave, which corresponds to photoreceptor activation, and b-wave, which arises from the signal being propagated in the retina, response amplitudes were significantly higher than in vehicle-treated control animals (Fig. 1C–E). Spectral-domain optical coherence tomography (SD-OCT) and histological analyses were used to examine the retinal architecture and measure the outer nuclear layer (ONL) thickness of P23H-1 rats. SD-OCT and histological measurements showed increased thickness of the ONL in HSP990-treated animals (Fig. 1F and G).

**Figure 2.**
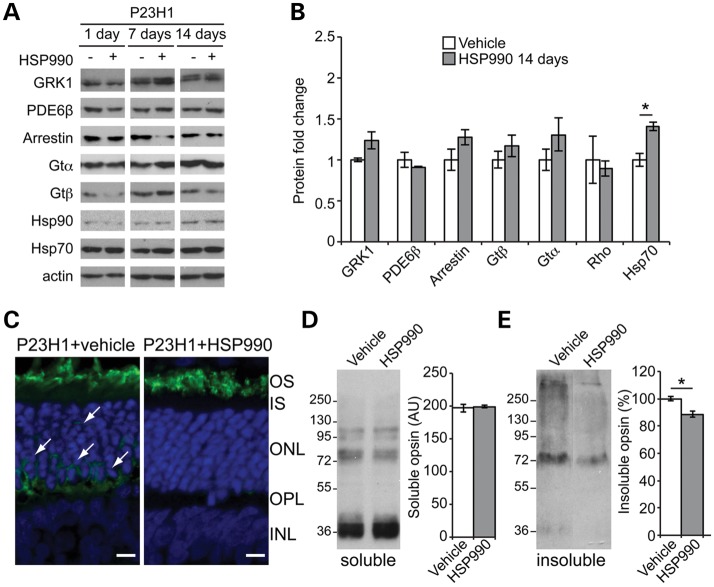
HSR induction and reduced aggregation in the P23H-1 rat retina following HSP990 treatment. (**A**) Western blots of P23H-1 rat retinae treated with a single dose of vehicle or HSP990 at 21 days of age after 1, 7 or 14 days, as indicated. (**B**) Quantification of expression levels of phototransduction proteins and Hsp70 in P23H-1 rat retina relative to levels of actin, 14 days after HSP990 administration. Western blots were subjected to densitometric analyses. Fold expression of each protein was calculated for HSP990 relative to vehicle. *P ≤ 0.05, values are means ± SEM, *n* ≥ 3. (**C**) Representative images of ONL from HSP990 or vehicle-treated P23H-1 animals with rhodopsin stained in green and nuclei in blue with DAPI. Cell body staining is arrowed. Scale bars: 10 μm. Representative western blots and densitometric quantitation of soluble (**D**) and insoluble (**E**) rhodopsin fractions revealed a reduction only in the insoluble fraction following HSP990 treatment. The position of molecular-weight markers is indicated on the left in kDa for the blots. *P < 0.05, ANOVA values are mean ± SEM (*n* ≥ 3 per treatment group).

Retinal protein expression was compared at different time points (1 day, 7 days and 14 days postdosing). Hsp70 levels in P23H-1 HSP990-treated rats were significantly increased at all time-points with a peak at 7 days postadministration (Fig. 2A, B and Supplementary Material, Fig. S1), whereas Hsp90 levels remained unchanged (Fig. 2A). HSP990 treatment had no significant effect on a range of phototransduction protein levels at the time points analyzed (Fig. 2A, B and Supplementary Material, Fig. S1). Immunohistochemistry confirmed the correct localization of rhodopsin in the outer segment (OS) in HSP990-treated P23H-1 rats with less cell body rhodopsin staining in the ONL compared with vehicle-treated controls (Fig. 2C). Interestingly, although the amount of soluble rhodopsin was unchanged (Fig. 2D), HSP990 treatment led to a significant reduction of sedimentable, insoluble rhodopsin (Fig. 2E), suggesting a reduction in rhodopsin aggregation that correlated with improved photoreceptor function and survival.

### Pharmacological manipulation of R135L rhodopsin

**Figure 3.**
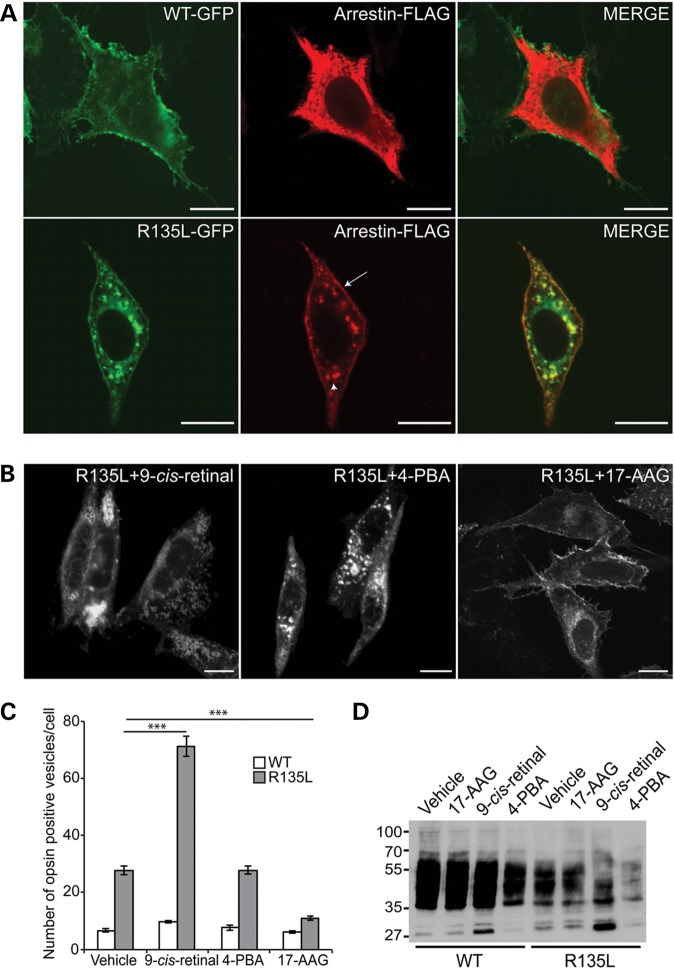
Pharmacological manipulation of R135L rhodopsin. (**A**) Subcellular distribution and trafficking of WT-GFP and R135L-GFP rod opsin (green) in SK-N-SH neuroblastoma cells co-transfected with visual arrestin-FLAG (red). WT rod opsin mainly decorated the PM and visual arrestin remained in the cytoplasm. R135L rod opsin mutant recruited and translocated cytosolic visual arrestin to the PM (arrow) and the endocytic compartments (arrowhead). Scale bars: 10 μm. (**B**) Fluorescence microscopy of R135L-GFP rod opsin and treated with 10 μm 9-*cis*-retinal, 10 mm 4-PBA and 1 μm 17-AAG. Scale bars: 10 μm. (**C**) Cell counts of intracellular vesicle incidence in cells expressing WT-GFP (open) or R135L-GFP (grey) following treatment with 9-*cis*-retinal, 4-PBA or 17-AAG for 18 h. Values are mean ± SEM. ***P < 0.0001, Student's *t*-test *n* = 3. (**D**) Western blot with 1D4 of untagged WT and R135L rod opsin after treatment with 9-*cis*-retinal, 4-PBA or 17-AAG for 24 h. The position of molecular-weight markers is indicated on the left in kDa.

To date, no pharmacological treatments have been reported that can ameliorate the phenotype of the Class III rod opsin mutant R135L. As previously described (14), R135L was constitutively internalized from the PM to endocytic vesicles that accumulated intracellularly in SK-N-SH neuroblastoma cells (Fig. 3A and Supplementary Material, Fig. S2). By contrast, WT rod opsin was present mainly on the PM (Fig. 3A and Supplementary Material, Fig. S2). Western blot analysis showed that the expression level of the R135L rod opsin mutant was reduced compared with the WT protein, but had the same electrophoretic mobility and glycosylation pattern after treatment with Endoglycosidase H (EndoH) or Peptide-*N*-Glycosidase F (PNGaseF) (Supplementary Material, Fig. S2B). This indicates that the mutant protein progressed through the secretory pathway from the endoplasmic reticulum (ER) to the Golgi and was not retained in the ER like the Class II mutant P23H (Supplementary Material, Fig. S2C). In cells co-transfected with visual arrestin and WT rod opsin, visual arrestin localized in the cytoplasm and did not co-localize with rod opsin (Fig. 3A). However, co-transfection of R135L rod opsin led to the recruitment and translocation of visual arrestin from the cytoplasm to the PM and endocytic compartments where it co-localized with R135L rod opsin (Fig. 3A). P23H rod opsin was retained in the ER and did not alter arrestin localization or lead to intracellular vesicle formation (Supplementary Material, Fig. S2C).

**Figure 4.**
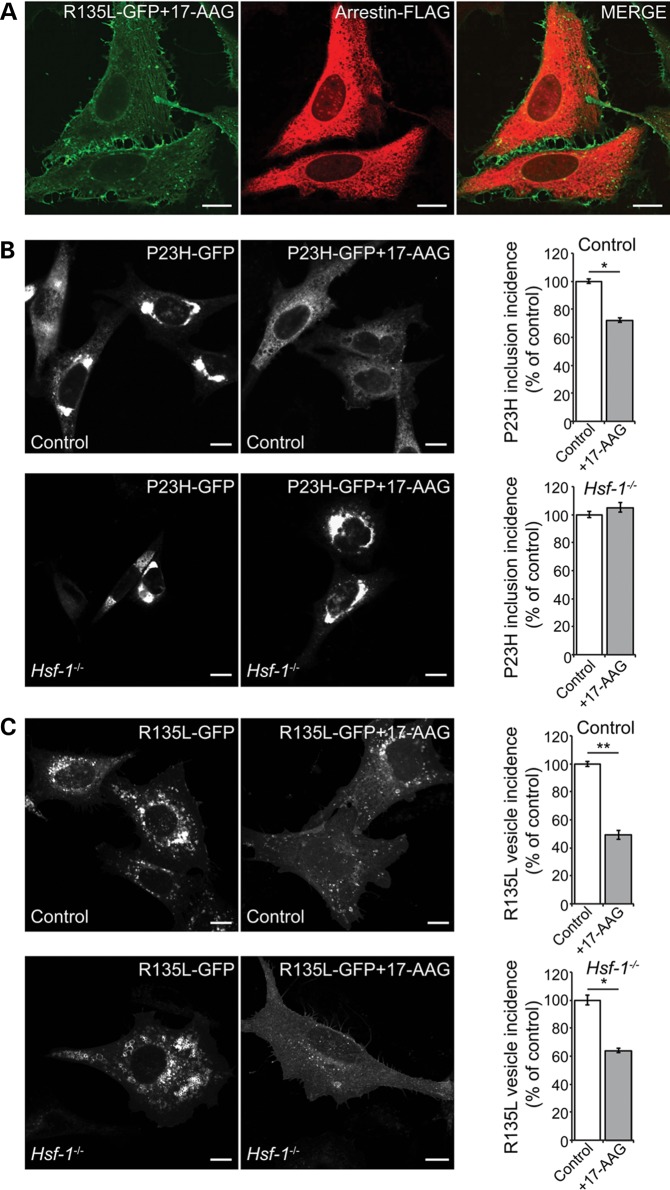
Hsp90 inhibition affects P23H and R135L by different mechanisms. (**A**) Subcellular distribution and trafficking of rod opsin in SK-N-SH cells co-transfected with R135L-GFP rod opsin (green) and arrestin-FLAG (red) and treated with 17-AAG (1 μm) for 24 h. (**B**) Representative images of P23H-GFP expressing control (upper panels) and *Hsf-1*^−/−^ MEFs (lower panels) treated with vehicle or 17-AAG (0.5 μm) for 20 h as indicated (left panels). At least 600 cells were scored for the presence of inclusions in each condition and the inclusion incidence was normalized to vehicle-treated inclusion incidence (right panels). Values are mean ± SEM. *P < 0.05, Student’s *t*-test. (**C**) Representative images of R135L-GFP expressing control (upper panels) and *Hsf-1*^−/−^ MEFs (lower panels) treated with vehicle or 17-AAG (0.5 μm) for 20 h, as indicated (left panels). Cells were scored for large intracellular accumulations of R135L-positive vesicles and the values normalized to vehicle-treated MEFs (right panels). At least 600 cells were scored in each condition. Values are mean ± SEM. *P < 0.05, **P < 0.01, Student’s *t*-test. Scale bars: 10 μm.

Several different pharmacological approaches can affect P23H rod opsin traffic or aggregation in cells (12). Therefore, the most effective compounds from each approach (9-*cis*-retinal, 4-phenyl butyric acid (4-PBA) and 17-AAG) were tested for an effect on R135L rod opsin. 11- and 9-*cis* retinal can act as pharmacological chaperones to stabilize near native rod opsin conformations and improve P23H rod opsin folding and trafficking (12,19,20). Treatment of cells expressing R135L rod opsin with 9-*cis*-retinal enhanced the endocytosis of the mutant protein and shifted the protein from the PM to the intracellular vesicles (Fig. 3B and C), whereas treatment with the low-molecular-weight fatty acid and kosmotrope, 4-PBA that reduces P23H rod opsin aggregation (12), did not affect R135L rod opsin localization (Fig. 3B and C). In contrast, treatment of cells expressing R135L rod opsin with the Hsp90 inhibitor 17-AAG reduced the number of intracellular vesicles (Fig. 3B and C). Soluble fractions of cells transfected with R135L rod opsin and treated with 9-*cis*-retinal, 17-AAG or 4-PBA for 24 h were analyzed by western blotting (Fig. 3D). The levels of soluble R135L protein were not affected after treatment with 17-AAG, while treatment with 4-PBA reduced the expression of both WT and the R135L rod opsin. Treatment with 9-*cis*-retinal stimulated an increase of a low-molecular-weight species at ∼27 kDa for both proteins, potentially corresponding to cleavage of the opsin *N*-terminal segment (21). Cells co-transfected with R135L rod opsin and arrestin and treated with 17-AAG for 24 h showed a reduction in R135L-arrestin co-localization (Fig. 4A), restoring the normal arrestin localization to the cytoplasm and reducing the number of R135L rod opsin positive intracellular vesicles (Fig. 4A).

### HSF-1 dependence of Hsp90 inhibition effects on P23H and R135L rod opsin

The protective effect of Hsp90 inhibition on P23H rhodopsin correlated with an increase in heat shock protein expression, so we tested if the effect was dependent on HSF-1. Control and *Hsf-1* null (22) mouse embryonic fibroblasts (MEFs) were transfected with P23H-GFP rod opsin and inclusion incidence was assessed as a surrogate marker of protein aggregation. As previously described (12), 17-AAG treatment led to a significant reduction in inclusion incidence in control cells (Fig. 4B). In contrast, there was no reduction in inclusions in the *Hsf-1*^−/−^ cells treated with 17-AAG (Fig. 4B), confirming that the protective effect of Hsp90 inhibition on P23H is dependent on activation of the HSR.

As previously observed in SK-N-SH cells, the intracellular accumulation of R135L-GFP rod opsin in control MEFs was reduced by 17-AAG treatment. Hsp90 inhibition was also effective at reducing intracellular R135L in *Hsf-1*^−/−^ cells (Fig. 4C), showing that this effect is independent of HSF-1. These data highlight that different mechanisms underlie the effect of Hsp90 inhibition on P23H and R135L rod opsin and suggest that the rescue of R135L is likely to be the result of targeting another Hsp90 client protein.

### The rescue of R135L rod opsin by Hsp90 inhibition is dependent on GRK activity

**Figure 5.**
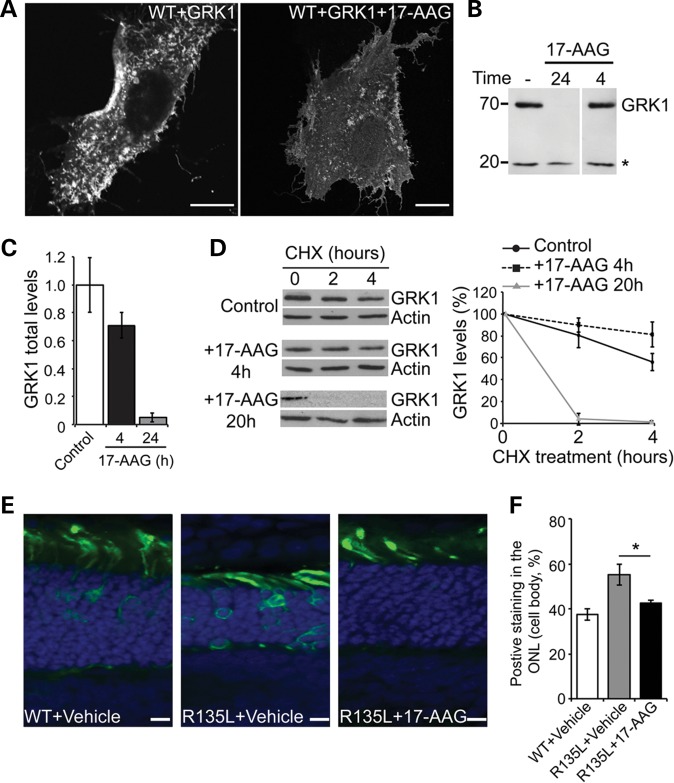
Hsp90 inhibition blocks R135L:arrestin recruitment through GRK1 (**A**) SK-N-SH cells co-transfected with WT-GFP rod opsin and FLAG-GRK1 and treated with 17-AAG (1 μm) or vehicle for 24 h. Scale bars: 10 μm. (**B**) Western blot of FLAG-GRK1 expression following 17-AAG treatment. SK-N-SH cells were transfected with FLAG-GRK1 and treated with 17-AAG (1 μm) for 24 or 4 h as indicated. Ten micrograms of soluble protein were resolved and detected using anti-FLAG mAb. Asterisk highlights a non-specific band used as a loading control. (**C**) Quantification of total FLAG-GRK1 levels, normalized relative to loading control, after incubation with 17-AAG for the indicated times. Values are mean ± SEM (*n* > 6). (**D**) Degradation of GRK1 in the presence of 17-AAG. Left panel, western blot for FLAG-GRK1 and actin of SK-N-SH cell lysates treated with 17-AAG for the indicated times prior to addition of CHX (50 μg/ml) for the indicated times. Exposures have been adjusted so that time 0 is approximately equivalent. Right panel shows quantification of GRK1 levels normalized to time 0. Values are mean ± SEM (*n* ≥ 3). (**E**) *In vivo* electroporation of WT and R135L rod opsin. WT-GFP or R135-GFP (green), as indicated, was injected subretinally with vehicle or 17-AAG (20 mg/ml) and electroporated in neonatal SD rats. Eyes were analyzed 16 days postelectroporation. Nuclei were stained with DAPI. Scale bars: 10 μm. (**F**) Quantitation of over 100 transfected cells (*n* = 4) showed WT-GFP was mainly present in the ROS, whereas R135L-GFP in the presence of vehicle was observed throughout the photoreceptor cell layer. Treatment with 17-AAG partially shifted the mutant protein to the ROS and reduced cell body staining. * *P* < 0.05, Student's *t*-test.

Hsp90 plays a general role in regulating G-protein-coupled receptor kinase (GRK) maturation, and GRK2, GRK3, GRK5 and GRK6 are stabilized by interaction with Hsp90 (23). We therefore hypothesized that treatment with 17-AAG might disrupt the interaction between the R135L mutant and arrestin by inhibiting Hsp90 function in kinase maturation and thereby alter the phosphorylation status of R135L rod opsin. Overexpression of rhodopsin kinase (GRK1) leads to constitutive phosphorylation of WT rod opsin (14). Similarly, when WT rod opsin was co-expressed with GRK1, we observed staining of WT rod opsin in the PM and re-localization to intracellular vesicles, mimicking the R135L phenotype (Fig. 5A). Treatment of cells co-expressing WT rod opsin and GRK1 with 17-AAG for 24 h prevented accumulation of intracellular vesicles and restored the localization of rod opsin protein mainly to the PM (Fig. 5A). These data suggest that Hsp90 inhibition has an effect on GRK1, and most likely other GRKs, rather than directly on the mutant rod opsin protein. Treatment with 17-AAG for 24 h led to a significant reduction of GRK1 levels (>90%), whereas after 4 h GRK1 protein levels were only mildly reduced (∼30%) (Fig. 5B and C). These data suggest that Hsp90 is required for GRK1 synthesis, but not GRK1 stability once the protein has been correctly folded. To test this hypothesis, GRK1 levels were examined following cycloheximide (CHX) treatment to block protein synthesis and reveal the rates of protein degradation. After 20 h of 17-AAG treatment GRK1 was rapidly degraded (Fig. 5D), with a half-life of <2 h. In contrast, after 4 h of 17-AAG treatment the remaining GRK1 was relatively stable, with a half-life in excess of 6 h (Fig. 5D). These data confirm that Hsp90 is required for the stability of newly synthesized GRK1, but once GRK1 has been folded Hsp90 does not appear to be required for its stability.

### R135L rod opsin localization in photoreceptors is restored by Hsp90 inhibition

To confirm our *in vitro* studies, and test if Hsp90 inhibition had an effect on R135L mutant rod opsin *in vivo*, electroporation was used to introduce DNA constructs encoding WT-GFP and R135L-GFP rod opsin into the developing rat retina (14,24), plus vehicle or 17-AAG in the injection. Neonatal SD rats electroporated with WT-GFP and vehicle treated showed rod opsin mainly targeted to the rod photoreceptor OS, as expected (Fig. 5E). Mutant R135L-GFP also localized to the OS, but showed additional increased retention in the photoreceptor cell body, most likely corresponding to arrestin-mediated internalization (Fig. 5E and F) (14). R135L rod opsin injection in combination with 17-AAG resulted in a significant reduction of R135L rod opsin in the cell body (Fig. 5E and F). This result confirmed the *in vitro* data and showed that 17-AAG was able to restore R135L rod opsin localization to WT phenotype *in vivo*. In addition, Hsp90 inhibition seemed to enhance the vectorial transport of R135L mutant rod opsin to the OS by suppressing its endocytosis defect.

### Prolonged high dose Hsp90 inhibition reduces GRK1 and PDE levels *in vivo*

**Figure 6.**
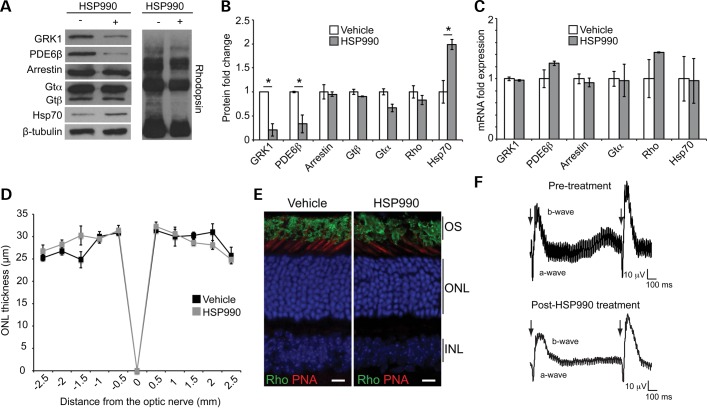
Systemic administration of HSP990 reduces GRK1 and PDE6 levels. (**A**) Western blots of 10 μg total retina protein for phototransduction proteins or Hsp70 from mice treated once every 3 days with HSP990 (80 mg/kg) for 10 days. (**B**) Expression levels of phototransduction proteins and Hsp70 in mouse retina normalized to β-tubulin. Fold expression of each protein was calculated following HSP990 treatment relative to vehicle. Values are mean ± SEM (*n* ≥ 3 per treatment group). *P < 0.05, Student's *t*-test. (**C**) RT–PCR of retinal cDNA to determine the fold induction of phototransduction and Hsp70 (*HSPA1*) genes following 10 days of HSP990 (80 mg/kg) treatment relative to expression of the vehicle-treated mice. Values are mean fold ± SEM (*n* ≥ 3 per treatment group). *P < 0.05, Student's *t*-test. (**D**) Spider plot showing ONL thickness of vehicle or HSP990 (80 mg/kg)-treated mice once every 3 days for 10 days. Values are mean ± SEM (*n* ≥ 3 per treatment group). (**E**) Retinal sections stained for rhodopsin with 1D4 antibody (green), cone OS with PNA (red) and DAPI to visualize nuclei (blue) showed rhodopsin localization in the ROS in both vehicle and HSP990-treated conditions. (**F**) Double-flash ERGs of mice pre- and post-HSP990 treatment. Recovery of the a- and b-waves was analyzed by delivering a double flash at different interval stimulus illumination (ISI). The traces displayed correspond to ISI(s) = 1 and show a good recovery of the a- and b-wave following HSP990 treatment. A representative trace of one animal is shown per treatment (*n* = 3 per treatment group).

Recent reports from oncology clinical trials have suggested that some Hsp90 inhibitors, such as 17-DMAG and AUY922, might lead to visual disturbances (25–27). Our data on the effect of 17-AAG on GRK1 suggest that some of the visual problems observed in patients could be on-target effects on phototransduction components. To test this hypothesis, HSP990 was given to control mice every 3 days for 14 days at close to the maximum tolerated dose, which was most likely to reveal any effect on phototransduction machinery biogenesis (Fig. 6A and B). HSP990-treated retinae showed a significant reduction in GRK1 levels, compared with vehicle-treated control (Fig. 6A and B). Interestingly, phosphodiesterase (PDE6β) levels were also reduced (70% reduction), but no significant decrease of arrestin, transducin and rhodopsin was observed (Fig. 6A and B). To confirm that these alterations were occurring post-transcriptionally, GRK1, PDE6β, arrestin, transducin α and rhodopsin mRNA levels were assessed by real-time quantitative PCR. No significant changes were observed in any of the genes assessed (Fig. 6C). These data suggest that inhibition of Hsp90 in the retina affects post-transcriptional levels of GRK1 and PDE6β as a result of chaperone deficiency.

To study any possible toxic effects of HSP990 on mouse retina, the retinae of these animals were processed for histological analysis. Photoreceptor survival was unaffected by HSP990 treatment (Fig. 6D). Moreover, HSP990-treated retinae exhibited retinal morphology that was indistinguishable from age-matched vehicle-treated animals, with rhodopsin localized in the OS (Fig. 6E). Retinal function was examined by ERG, and single-flash ERG responses of increasing intensity showed slightly reduced a-wave amplitudes in animals treated with HSP990 (Fig. 6F). A double-flash recording was used to study the recovery of phototransduction in photoreceptors. HSP990-treated mice exhibited the same complete a- and b-wave recovery as observed pretreatment (Fig. 6F); therefore, it is likely that the residual levels of PDE and GRK1 (20–30% of WT) are sufficient for photoreceptor function and survival.

## DISCUSSION

Mutations in rhodopsin result in distinct changes in the protein's cellular and biochemical properties that lead to RP through different mechanisms. Understanding the aspects of rhodopsin biology and associated molecular mechanisms, from protein biogenesis to receptor inactivation, is necessary for the development of novel effective therapeutic strategies to mitigate this currently untreatable retinal degenerative disease.

Class II misfolding mutants in rod opsin, such as P23H, are thought to result in photoreceptor cell death through a combination of gain-of-function and dominant-negative mechanisms (11). The possible gain-of-function effects include ER retention and unfolded protein response (UPR) activation, proteasome inhibition, protein instability and aggregation and dysregulated activation (11,28,29). The critical factors have still not been fully defined, but successful protection against P23H rhodopsin-mediated cell death has been reported with overexpression of the molecular chaperone BiP (HSPA5) (30,31) or treatment with curcumin and TUDCA (32,33), which might act to reduce protein misfolding stress and protein aggregation. Here, we observed protection against P23H rhodopsin-mediated disease with Hsp90 inhibition that correlated with the induction of molecular chaperones via activation of HSF-1 and reduced rhodopsin aggregation, suggesting that HSR manipulation could be important for combatting the gain-of-function properties of Class II rod opsin mutants. Targeting imbalances in photoreceptor proteostasis might have broader application. For example, 17-AAG treatment after transient blood retinal barrier permeabilization with claudin 5 RNAi successfully prevented photoreceptor degeneration in an RP10 IMPDH mouse model, showing effective reduction of mutant R224P IMPDH aggregation and protection of ONL structure (17). Therefore, we believe that in the P23H model Hsp90 inhibition acts as a protective factor via enhanced activation of the cell stress machinery. This provides proof of principle that HSF-1 activation can protect photoreceptors from protein misfolding stress.

Rhodopsin phosphorylation is part of the normal inactivation pathway of the phototransduction cascade and leads to arrestin binding (34). The adRP rod opsin substitutions K296E and R135L, however, have been shown to be hyperphosphorylated, which can lead to altered arrestin binding which then recruits the endocytic adaptor protein AP-2, potentially leading to defects in subcellular traffic (14,35,36). We have shown that Hsp90 inhibition can rescue rod opsin mutant R135L-mediated recruitment of arrestin and mislocalization phenotype *in vitro* and *in vivo*, most likely by abolishing the hyperphosphorylation status of R135L. We hypothesized that this was not through a direct effect on R135L rod opsin as an Hsp90 client, but instead reveals a requirement for Hsp90 function for GRK1 synthesis. Overexpressing GRK1 with WT rod opsin led to the formation of intracellular vesicles and Hsp90 inhibition restored the WT phenotype, supporting the hypothesis that it is not a direct effect on the mutant rod opsin.

In *Drosophila*, inhibition of the formation of the rhodopsin:arrestin complex prevented photoreceptor degeneration (37). Thus, it is possible that prevention of this complex will also protect against degeneration in vertebrate retina. This hypothesis is supported by enhanced photoreceptor survival following expression of p44, which lacks the AP-2-binding element, to inhibit recruitment of AP-2 by the K296E:arrestin complex (36). There is no transgenic animal model for R135L rod opsin, but from the *in vivo* retinal R135L transduction data from Chuang *et al*. (14) and the data presented in this study, it can be inferred that the R135L mutant might cause cell death through the same mechanism. Therefore, Hsp90 inhibition mediated prevention of arrestin binding and endocytosis might protect against R135L rod opsin-mediated RP *in vivo*.

A range of Hsp90 inhibitors have now been developed with different affinities and bioavailability (3). Importantly, several have been in oncology clinical trials so their pharmacokinetic profile and side effects are being identified, such that they could potentially be applied to RP and other neurodegenerative disease with prior knowledge of the risks and benefits. In order to have a beneficial effect on retinal degeneration a systemically administered inhibitor would need to cross the blood retinal barrier (e.g. HSP990). However, there is a risk of systemic side effects of Hsp90 inhibition, especially over a long course of treatment. Alternatively, those inhibitors that do not cross the blood retinal barrier efficiently (e.g. 17-AAG) would need be used in conjunction with a technique that transiently permeabilizes the blood retinal barrier (17), or be administered by intravitreal or subretinal injection (e.g. Fig. 5). Topical delivery might reduce the risk of systemic side effects of Hsp90 inhibition and this approach has been successfully used for retinal neovascularization therapy with anti-VEGF biological agents, but the repeated long-term dosing needed for RP therapy means that this is unlikely to be an attractive clinical option. A treatment regime could be based on a repeated low dose administration that was just sufficient to repeatedly stimulate the HSR. However, the ability to induce the HSR in the brain can also be affected by epigenetic changes related to disease and aging (8), so it is possible that this protective effect might not be sustained over the many years required to prevent cell death in the retina or in other forms of neurodegeneration. This will need to be explored further with extended treatments in slower models of retinal degeneration. Furthermore, the direct effects of sustained Hsp90 inhibition on photoreceptor function must be considered.

Chronic systemic Hsp90 inhibition led to a post-transcriptional reduction in GRK1 and PDE subunits in the retina while other phototransduction components such as rhodopsin, arrestin and transducin were unaffected. These results suggest that Hsp90 is an essential chaperone for the biogenesis of GRK1 and PDE6β. Reduction of these phototransduction components can affect vision. Patients lacking GRK1 or arrestin suffer from Oguchi disease, a form of stationary night blindness, which is not characterized by rod cell death (38,39). Therefore, loss of GRK1 itself might not lead to rod cell death, but would lead to rod and cone dysfunction, as cone GRK7 is also likely to be an Hsp90 client protein. The co-chaperones that function with Hsp90 in GRK biosynthesis are not known, but might include Cdc37, which assists Hsp90 in the maturation of many kinase clients (40). Furthermore, Hsp90 inhibition reduced the level of PDE. These data support the hypothesis that Hsp90 may assist the photoreceptor chaperone AIPL1 in PDE biogenesis. Mutations in AIPL1 cause Leber congenital amaurosis (LCA), a severe early onset retinopathy (41) and AIPL1 functions as a chaperone specific for PDE biosynthesis (42,43). In addition, AIPL1 is a co-chaperone for Hsp90 (44). Therefore, these data would support a role for both Hsp90 with AIPL1 in a chaperone heterocomplex that is essential for PDE biogenesis and maturation. The other chaperones and co-chaperones that are involved in PDE biogenesis remain to be identified, but might include Hsp70 as that can also bind AIPL1 (44).

In a recent clinical trial for advanced solid tumors with AUY922, a potent second-generation, non-geldanamycin isoxazole Hsp90 inhibitor, 43% of the patients reported grades 1–3 visual symptoms, including night blindness, photopsia, blurred vision and visual impairment (27). These visual symptoms, in particular night blindness and visual impairment, could be due to reductions in PDE and GRK1. Fortunately, all the visual symptoms were reversible when the drug was discontinued. We did not observe retinal toxicity with HSP990 in mice as the ONL thickness and mRNA transcript levels of photoreceptor proteins were unaffected in animals treated with a high dose of HSP990. Moreover, there were only small effects on the ERG at these doses of HSP990. The reduced a-wave amplitude presumably relates to the lower levels of PDE, but dark adaptation appeared unaffected. It is likely that the residual levels of PDE and GRK1 (20–30% of vehicle treated) were sufficient for photoreceptor function and survival. It is possible that greater Hsp90 inhibition in the retina by higher affinity Hsp90 inhibitors with increased retinal availability could lead to larger reductions in GRK1 and PDE, which would have a more pronounced effect on photoreceptor function. Further reductions in PDE could affect photoreceptor survival and be irreversible, as complete loss of PDE is toxic to photoreceptors (43). This needs to be considered for each Hsp90 inhibitor and might need to be tested empirically as it could have important implications for the use of Hsp90 inhibitors in the treatment of cancer or neurodegeneration. However, our data show that Hsp90 can be inhibited substantially in the retina without toxic effects. Collectively, the data show that Hsp90 has multiple roles in the retina and that the use of Hsp90 inhibitors can be potentially protective against retinal degeneration, but their possible adverse effects on visual function also need to be considered.

## MATERIALS AND METHODS

### Materials

FLAG-GRK1 plasmid was a gift from Dr Ellen Weiss (University of North Carolina, NC, USA). Visual Arrestin-FLAG plasmid was a gift from Professor Vsevolod Gurevich (Vanderbilt University Medical Center, Nashville, TN, USA). Lipofectamine and Plus reagent were purchased from Invitrogen. Protease Inhibitor Cocktail, 9*-cis-*retinal, 17-allylamino-17-demethoxygeldanamycin (17-AAG), 4-PBA and 4′,6-diamidino-2-phenylindole dihydrochloride (DAPI) were purchased from Sigma. HSP990, a 2-amino-7,8-dihdro-6H-pyrido[4,3-d]pyrimidin-5-one compound, was obtained from Novartis (Basel, Switzerland). EndoH and PNGaseF were from New England Biolabs. Rhodamine-labeled peanut agglutinin (PNA: 1:200) was from Vector Laboratories. Rod opsin constructs, untagged rod opsin in pMT3 and rod opsin-GFP were described previously (19). 1D4 mouse mAb against rod opsin was a gift from Professor Robert Molday (University of British Columbia, Vancouver, Canada) (1.33 mg/ml; 1:1500 for immunoblotting and for immunohistochemistry, 1:5000 for immunocytochemistry). Hsp40 mouse mAb (1:600) was a gift from Professor Boris Margulis (Institute of Cytology of the Russian Academy of Sciences, St. Petersburg, Russia). FLAG (clone M2) mouse mAb (1:1000), Hsp60 (clone LK1) mouse mAb (1:400), β-tubulin mouse mAb (1:5000) and actin mouse mAb (1:2000) were from Sigma. GRK1 (clone G8) mouse mAb (1:1000) and HSF-1 rat mAb (1:1000) were from Abcam. Hsp70 mouse mAb (1:1000) and Hsp90 rat mAb (1:5000) were from Stressgen. PDE6β rabbit pAb (1:1000) and Arrestin rabbit pAb (1:1000) were from ThermoScientific. PDE6β mouse mAb (1:1000) for rat tissue, transducin α rabbit pAb (1:1000) and transducin β rabbit pAb (1:1000) were from Santa Cruz. Goat anti-mouse Alexa Fluor 488 or 594 secondary antibodies conjugated IgGs (1:1000) were from Invitrogen. Goat anti-mouse (1:50000), anti-rabbit (1:30000) or anti-rat (1:30000) secondary antibodies conjugated with horseradish peroxidase were from Pierce.

### Animals

All procedures were conducted according to the Home Office (UK) regulations under the Animals (Scientific Procedures) Act of 1986 and with local UCL Institute of Ophthalmology, London, UK ethics committee approval. Three types of animals were used wild-type C57Bl6 mice, wild-type Sprague Dawley rats (SD) and P23H-1 heterozygous rats kindly provided by Professor Matt LaVail (University of California San Franscisco, San Franscisco, CA, USA). All animals were housed under a 12:12 light dark cycle, with food and water available *ad libitum*.

### HSP990 dosing

HSP990 was formulated using 2% methyl cellulose diluted with 9 volumes of saline solution (water with 0.9% NaCl). The HSP990 vehicle mixture was sonicated at high frequency in a water bath and mixed thoroughly to form a uniform suspension. Compound or vehicle alone was administered to mice or rats by oral gavage. For single-dose experiments, female rats were treated with 1 mg/kg and male rats with 5 mg/kg because of sex differences in sensitivity. For prolonged doses mice were treated with 80 mg/kg every 3 days.

### Immunocytochemistry

SK-N-SH cells were maintained and transfected as described (12). Twenty-four hours after transfection, cells were fixed with 4% paraformaldehyde (PFA) for 15 min and permeabilized in 0.5% Triton X-100 for 10 min. Non-specific binding was blocked using 3% BSA, 10% serum of the secondary antibodies species in PBS for 1 h. *Hsf-1*^−/−^ MEFs were derived from the C;129-Hsf-1tm1Ijb/J strain of mice (22) from the Jackson Labs and were a gift from Professor Gillian Bates and Dr Andreas Neueder (King's College London, UK). Control MEFs have been previously described (45). 1 × 10^6^ MEFs were transfected with 4 μg of P23H-GFP or R135L-GFP rod opsin plasmids using the Nucleofector kit from Lonza (Slough, UK). Cells were treated with 17-AAG (0.5 μm) or vehicle treated for 20 h and fixed with 4% PFA. Images were taken using a Carl Zeiss LSM 710 laser-scanning confocal microscope. The images were exported from LSM Browser and prepared using Adobe Photoshop and Illustrator CS4. Cell morphology studies scored the predominant localization of rod opsin on the PM, ER, inclusions or vesicles as a percentage of total transfected cells. At least four fields of ∼100 cells were counted for each condition.

### Immunohistochemistry

At the defined time points, animals were sacrificed, and the eyes were collected for histology. Eyes were fixed overnight in 4% paraformaldehyde in PBS, and left in a 30% sucrose solution for cryoprotection. Eyes were then frozen in OCT compound (VWR) using a dry ice/acetone slurry and stored at −80°C prior to cryosectioning at 10 μm onto Superfrost plus slides (VWR). Cryosections were incubated with primary antibodies 1D4 and PNA (1:200) and visualized with anti-mouse goat Alexa Fluor 488 conjugated IgGs. Outer nuclear layer thickness measurements were made on digital images of stained cryosections, every 500 μm from the optic nerve outwards for both the inferior and superior hemisphere.

### Immunoblotting

SK-N-SH transfected cells were lysed for 15 min at 4°C in 1% *n*-dodecyl-β-d-maltoside (DM) buffer with 2% protease inhibitor cocktail in PBS. For deglycosylation reactions, 15 µg total protein in DM soluble cell lysate was digested with EndoH or PNGase F. Digestions were carried out overnight at 37°C before resolving by SDS–PAGE. Frozen mouse or rat retinae were homogenized in ice cold RIPA buffer with 2% protease inhibitor cocktail. Protein concentration was determined by BCA assay (ThermoScientific) and 10 µg protein was added to 2× Laemmli loading buffer before SDS–PAGE and western blot. For the differential sedimentation assay, 350 µl of rat retinal lysate was centrifuged at 100 000*g* 10°C for 30 min resulting in supernatant-1 (S1, ‘soluble’) and pellet-1 (P1) fractions. An aliquot of S1 fraction was mixed with 4× SDS–PAGE sample buffer for future analysis while P1 was washed with 200 µl of RIPA buffer and reconstituted in RIPA buffer containing 5% SDS by sonication. This was centrifuged at 225 000*g* for 60 min at 20°C resulting in P2 and S2 fractions accordingly. The ‘insoluble’ P2 fraction was reconstituted in 2× SDS–PAGE sample buffer by sonication prior to analysis by western blotting. To assess protein degradation, SK-N-SH cells were treated with CHX (50 μg/ml) (Sigma, Poole, UK) 20 h after transfection for 2 or 4 h. Samples were analyzed by western blotting. Immunodetection of the proteins of interest was carried out using the primary and secondary antibodies described in Materials and Methods. Western blot densitometry was performed using ImageJ. Developed films were scanned, and the average pixel density for each band was measured.

### Electroretinography

Scotopic ERG was performed as described in Coffey *et al.* (46). Animals were dark-adapted overnight and anesthetized with ketamine/xylazine intraperitoneally (i.p.). Procedures were carried out under red-light conditions. Pupils were dilated with topical 1% tropicamide and 2.5% phenylephrine hydrochloride. ERG was carried out via platinum loop electrodes on the cornea and an indifferent platinum electrode in the scalp of the subjects. A platinum earth electrode was placed in the back of the animal. The animal was placed on a heated pad (37°C) in a screened light-tight box for recording. Flash stimuli (10 μs to 1 ms duration, repetition rate 0.1–1 Hz) were presented via an LED stimulator (log intensity −6 to + 2.7) under scotopic conditions.

### Optical coherence tomography

SD rats were imaged using a modified Spectralis OCT (Heidelberg Engineering, Heidelberg, Germany). Measurements were recorded for whole and individual retinal layer thickness in four regions (temporal, superior, nasal and inferior) around the optic nerve head. After manual segmentation of each layer, ONL thickness was measured with Adobe Photoshop.

### Electroporation

DNA encoding WT-GFP and R135L-GFP and pRho dsRed in fast green 0.1% were injected into the subretinal space of neonatal SD rats, followed by electroporation as described (24,47).

### RNA extraction, reverse transcription and real-time PCR

Retinae were dissected and total RNA extraction was performed using an RNeasy Mini Kit (Qiagen, Crawley, UK). cDNA synthesis, Invitrogen Super Script III First-Strand Synthesis System reversed transcription. RT–PCR was carried out using the ABI700HT (Applied Biosystems). The relative quantification of the genes of interest was performed in triplicate using three biological replicates according to the comparative method. Specific amplification of gene transcripts was achieved with the following primers: *HSPA1A* (Hsp70) F: TGGTGCAGTCCGACATGAAG, R: GCTGAGAGTCGTTGAAGTAGGC; *GRK1* F: CGGGGCAGTTTTGACGGAA, R: AGCTGAGGTTGTCACGGAGA; *PDE6β* F: GCAGCACTTTTTGAACTGGTG, R: CATTGCGCTGGCGGTACATA; *Arrestin* F: GCCTGCGGGAAGACCAATAAA, R: GGTCAGGGTGACATACACCTT; *Transducin α* F: GATGCCCGCACTGTGAAAC, R: CCAGCGAATACCCGTCTTG; *Rhodopsin* F: CCCTTCTCCAACGTCACAGG, R: TGAGGAAGTTGATGGGGAAGC. An internal reference (*GAPDH*) was used to normalize the transcripts: F: GCCATCAACGACCCCTTCAT, R: ATGATGACCCGTTTGGCTCC. The reaction was set up using the SYBR green Mix Hi-Rox (PCR Biosystems). The data were analyzed with DART PCR software (48).

## SUPPLEMENTARY MATERIAL

Supplementary Material is available at *HMG* online.

## FUNDING

This work was supported by the Wellcome Trust (grant number 092621); RP Fighting Blindness (grant numbers GR563; GR580); the Big Lottery Fund (grant number C170A106) and by Fundación Ramón Areces. Funding to pay the Open Access publication charges for this article was provided by UCL and the Wellcome Trust.

## Supplementary Material

Supplementary Data
