# Supplementary material for: Hsp90 inhibition protects against inherited retinal degeneration
Source: Hum Mol Genet. 2013 Dec 2;23(8):2164–75. doi: 10.1093/hmg/ddt613 (PMC3959821; doi:10.1093/hmg/ddt613)
Supplement: Supplementary Data [file supp_23_8_2164__index.html]

Hsp90 inhibition protects against inherited retinal degeneration — Hsp90 inhibition protects against inherited retinal degeneration — Supplementary Data 

# Hsp90 inhibition protects against inherited retinal degeneration

## Supplementary Data

Supplementary Data

**Files in this Data Supplement:**

- Supplementary Data - Pdf file
