# Supplementary material for: Hsp90 inhibition protects against inherited retinal degeneration
Source: Hum Mol Genet. 2013 Dec 2;23(8):2164–75. doi: 10.1093/hmg/ddt613 (PMC3959821; doi:10.1093/hmg/ddt613)
Supplement: Supplementary Data [file supp_ddt613_ddt613supp.pdf]

Hsp90 inhibition protects against inherited retinal degeneration

Mònica Aguilà, Dalila Bevilacqua, Caroline McCulley, Nele Schwarz, Dimitra Athanasiou, Naheed Kanuga, Sergey S. Novoselov, Clemens A.K. Lange, Robin R. Ali, James W. Bainbridge, Carlos Gias, Peter J. Coffey, Pere Garriga and Michael E. Cheetham

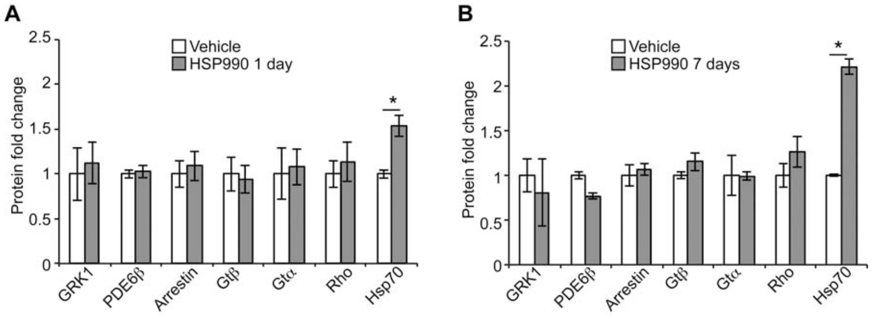

Supplementary material Figure 1. ***Hsp70 induction in P23H-1 retina following a single dose of HSP990.*** Quantification of expression levels of phototransduction proteins and Hsp70 in P23H-1 rat retina relative to levels of actin 1 day (**A**) and 7 days (**B**) after HSP990 administration. Western blots were subjected to densitometric analyses. Fold expression of each protein was calculated for HSP990 relative to vehicle. Values are mean ± SEM (n ≥ 3 per treatment group). *P<0.05 Student's *t* test.

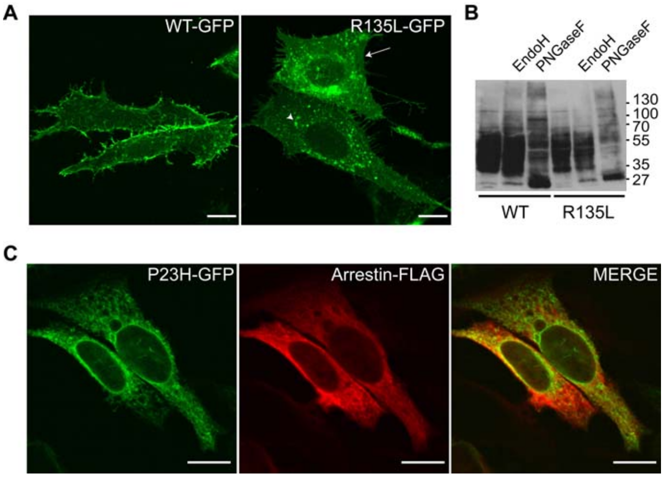

Supplementary material Figure 2. ***The R135L mutation stimulates rod opsin endocytosis.*** (**A**) GFP fluorescence in SK-N-SH cells transfected with WT-GFP and R135L-GFP rod opsin. WT-GFP was predominantly targeted to the PM whereas R135L-GFP mutant was targeted to the PM (arrow) and to the endocytic compartments (arrow head). (**B**) Western blot of untagged WT and R135L rod opsin with 1D4 antibody. The electrophoretic mobility of different glycoforms of opsin were determined empirically following EndoH and PNGase F digestion, as indicated. The position of molecular weight markers is indicated on the right. (**C**) Subcellular distribution and trafficking of P23H-GFP rod opsin in SK-N-SH cells co-transfected with visual Arrestin-FLAG. There was no overlap between P23H-GFP in the ER and arrestin in the cytoplasm, highlighting the difference between class II and class III rod opsin mutants. Scale bars 10 µm.
